# Hydroxysafflor Yellow A Regulates SIRT1-FOXO3-BNIP3 Signaling Pathway to Promote Mitophagy: A Novel Therapeutic Strategy for Myocardial Ischemia-Reperfusion Injury

**DOI:** 10.3390/nu18111780

**Published:** 2026-05-31

**Authors:** Dongdong Meng, Wencong Xia, Feng Tian, Qi Huang, Chaowen Ge, Ning Wang

**Affiliations:** 1 Anhui Province Key Laboratory of Chinese Medicinal Formula, Anhui University of Chinese Medicine, Hefei 230012, China; mddsci0214@163.com (D.M.); 18869418687@163.com (W.X.); tfeng201007@163.com (F.T.); 2 Institute for Pharmacodynamics and Safety Evaluation of Chinese Medicine, Anhui Academy of Traditional Chinese Medicine, Hefei 230012, China; 3 Anhui Province Key Laboratory of Bioactive Natural Products, Hefei 230012, China

**Keywords:** Hydroxysafflower Yellow A (HSYA), AC16 cell, myocardial ischemia-reperfusion injury (MIRI), mitophagy, SIRT1-FOXO3-BNIP3 signaling pathway

## Abstract

Background: Hydroxysafflor Yellow A (HSYA), the major bioactive component from *Carthamus tinctorius* L., exerts significant protective effects against myocardial ischemia-reperfusion injury (MIRI). Mitophagy is pivotal in the pathological process of MIRI, yet the specific molecular mechanism underlying HSYA-mediated mitophagy regulation remains unclear. Objective: This study aimed to investigate the association between HSYA treatment and mitochondrial autophagy in murine MIRI and to explore the potential mechanistic role of the SIRT1-FOXO3-BNIP3 signaling pathway using functional loss-of-function and rescue experiments. These findings may provide preliminary evidence supporting the clinical translational potential in MIRI therapy. Methods: Mouse myocardial ischemia-reperfusion injury (MIRI) model and oxygen-glucose deprivation/reoxygenation (OGD/R)-induced AC16 cardiomyocyte injury models were established. Metabolomics, molecular docking, and surface plasmon resonance (SPR) techniques were combined to screen the potential targets of HSYA. The SIRT1 inhibitor EX527 and SIRT1 siRNA were used to verify the underlying mechanism. Cardiac function, myocardial infarct size, mitochondrial function, the expression of autophagy-related proteins, and protein–protein interaction were detected and analyzed. Results: Compared with the MIRI group, HSYA significantly improved cardiac function in mice, as evidenced by increased left ventricular ejection fraction (LVEF) and left ventricular fractional shortening (LVFS) (*p* < 0.01), attenuated ST-segment elevation, and improved myocardial perfusion. HSYA also markedly reduced myocardial infarct size (*p* < 0.01) and serum levels of CK-MB, LDH, and cTnI (all *p* < 0.01) and ameliorated myocardial histopathological damage and mitochondrial ultrastructural integrity. Mechanistic studies revealed that HSYA significantly upregulated the expression of SIRT1, FOXO3, BNIP3, Beclin-1, and the LC3II/I ratio while downregulating p62 expression (*p* < 0.01), consistent with enhanced mitophagy-related activity. Furthermore, these protective effects were markedly attenuated upon SIRT1 inhibition or siRNA-mediated silencing, whereas HSYA intervention partially reversed these alterations. Additionally, co-immunoprecipitation (Co-IP) and pull-down assays demonstrated that HSYA promoted protein–protein interactions between SIRT1-FOXO3, FOXO3-BNIP3, and BNIP3-LC3B. Conclusions: These findings highlight that HSYA is associated with improved cardiac function, enhanced mitophagy-related activity, and upregulated SIRT1-FOXO3-BNIP3 signaling, providing robust experimental evidence for its clinical translational application in MIRI treatment.

## 1. Introduction

Coronary heart disease (CHD) is a disease caused by pathological changes of coronary atherosclerosis that lead to narrowing or blocking of the vascular lumen, poor blood flow of the heart’s coronary artery, insufficient blood supply to the myocardium, and hypoxia necrosis [1]. It is commonly referred to as “coronary heart disease” or “ischemic heart disease”. Among ischemic heart diseases, myocardial infarction (MI) is the leading cause of death [2]. Reperfusion of ischemic myocardium results in further pathological damage, such as cellular injury, arrhythmia, and microvascular obstruction [3]. The core mechanisms underlying this damage include oxidative stress, calcium overload, inflammatory response, and mitochondrial dysfunction, which are collectively termed MIRI [4]. Investigating the pathogenic mechanisms associated with MIRI and exploring potential therapeutic agents have become urgent and critical issues.

According to traditional Chinese medicine (TCM) theory, CHD falls into the categories of “thoracic obstruction” and precordial pain with cold limbs. Both Bi syndrome and pain syndrome in traditional Chinese medicine are both caused by obstruction of blood stasis in the heart meridian, which leads to pain [5,6]. The main treatment method of coronary heart disease is to promote blood circulation and remove blood stasis, and safflower is a representative Chinese medicinal material of traditional Chinese medicine for promoting blood circulation and removing blood stasis [7]. Traditional Chinese patent medicines and simple preparations such as safflower injection and safflower yellow injection, which are obtained from safflower extraction, can be used clinically for cardiovascular diseases, and HSYA is its main active ingredient [8]. HSYA exhibits extensive pharmacological effects against CHD. Our studies have demonstrated that HSYA crosses the blood-brain barrier (BBB) [9,10]. Furthermore, HSYA exerts a crucial therapeutic effect against cerebral ischemia by promoting angiogenesis in cerebral microvascular endothelial cells, thereby alleviating cerebral ischemia-reperfusion injury (CIRI) in rats [11,12]; it targets the ZBP1-166R locus to suppress MAVS signaling, thereby alleviating cerebral ischemia-reperfusion injury (CIRI) in mice [13]; it promotes microglial polarization to alleviate cerebral ischemia-reperfusion injury in mice [14]; HSYA improves myocardial energy metabolism after MIRI by activating the HIF-1α/SLC7A11/GPX4 signaling pathway to inhibit ferroptosis [15]. Additionally, HSYA alleviates myocardial ischemia-reperfusion injury in rats by promoting angiogenesis via the HIF-1α-VEGFA-Notch1 pathway [16]. Collectively, these findings highlight the great therapeutic potential of HSYA in the treatment of cardio-cerebrovascular diseases.

The occurrence of MIRI is often accompanied by mitochondrial damage and energy metabolism disorders. Mitochondrial impairment induces oxidative phosphorylation dysfunction, reduces ATP production, impairs cardiomyocyte function, and ultimately leads to a severe energy crisis [17]. To counteract cardiomyocyte apoptosis caused by mitochondrial damage, the body selectively degrades abnormal or damaged mitochondria to rectify mitochondrial dysfunction process commonly referred to as mitophagy. Therefore, regulating autophagy to improve mitochondrial function and maintain cardiac homeostasis is a potential therapeutic approach for MIRI [18,19]. Our studies have demonstrated that HSYA exerts therapeutic effects against cerebral ischemia in the brain by inhibiting autophagy via the PI3K/Akt/mTOR signaling pathway [20]. Relevant studies have demonstrated that inhibition of the SIRT1-FOXO3-BNIP3 signaling pathway could suppress mitophagy in hepatocellular carcinoma cells, thereby disrupting mitochondrial structure and function to exert an anti-tumor effect [21]. Meanwhile, HSYA can elevate the activity of SIRT1, which provides a technical basis for this experiment [14]. This suggests that the SIRT1-FOXO3-BNIP3 pathway could play a crucial role in regulating mitophagy.

SIRT1, a NAD+-dependent deacetylase, directly deacetylates and activates FOXO3, a transcription factor that upregulates BNIP3 expression. BNIP3 then functions as a mitophagy receptor by recruiting LC3 through its LIR motif, targeting damaged mitochondria for autophagic degradation. Recent studies have demonstrated that SIRT1 activation attenuates MIRI through autophagy induction [22] and that FOXO3-dependent mitophagy plays a protective role in diabetic cardiomyopathy [23]. However, several critical questions remain: (1) no study has investigated whether natural compounds can activate this pathway in MIRI; (2) the precise mechanism by which SIRT1 activation leads to BNIP3-dependent mitophagy in cardiomyocytes requires further validation; (3) most existing studies rely on either in vivo or in vitro approaches alone, lacking multi-level validation.

Based on the evidence reviewed above, we hypothesized that HSYA exerts cardioprotective effects against MIRI by modulating the SIRT1-FOXO3-BNIP3 signaling cascade and enhancing mitophagy-related mitochondrial quality control, thereby clearing damaged mitochondria and preserving cardiac function. To test this hypothesis, the present study was designed to (1) identify the potential molecular targets of HSYA using metabolomics and network pharmacology; (2) validate the binding affinity between HSYA and SIRT1 using SPR and molecular docking; (3) evaluate the cardioprotective effects of HSYA in a murine MIRI model and an OGD/R-induced AC16 cell model; and (4) elucidate the mechanism by which HSYA regulates SIRT1-FOXO3-BNIP3-mediated mitophagy using pharmacological (EX527, 3-MA) and small interfering RNA transfection (siRNA-SIRT1) approaches.

## 2. Materials and Methods

### 2.1. Experimental Approach

#### 2.1.1. Study Design

This study employed a multi-level, integrative experimental design comprising four sequential stages: (1) target identification: untargeted metabolomics and network pharmacology were used to identify differentially regulated metabolites and predict HSYA-SIRT1 interaction; (2) target validation: the direct binding affinity between HSYA and SIRT1 was validated using surface plasmon resonance (SPR) and molecular docking; (3) in vivo functional assessment: the myocardial ischemia/reperfusion injury (MIRI) model was established by ligating the left anterior descending coronary artery in mice to evaluate the cardioprotective effects of HSYA (5, 10, and 20 mg/kg) and investigate the SIRT1-FOXO3-BNIP3 signaling pathway, with EX527 (a specific SIRT1 inhibitor, 10 mg/kg) and 3-MA (an autophagy inhibitor, 10 mg/kg) used to confirm mechanism [14]; (4) in vitro mechanistic validation: an OGD/R-induced AC16 cell model with siRNA-mediated SIRT1 silencing was used to confirm the causal role of the pathway [16].

#### 2.1.2. Ethical Statement

A total of 100 male C57BL/6J mice (body weight: 22–25 g, Laboratory Animal Production License: SCXK2020-0005) were purchased from Henan Skbes Biotechnology Co., Ltd., Anyang China. All animal experiments were approved by the Experimental Animal Ethics Committee of Anhui University of Chinese Medicine (Approval No: AHUCM-mice-20250119) and conducted in accordance with the ARRIVE guidelines 2.0. The study protocol was designed to minimize animal suffering, with humane endpoints defined as (1) severe respiratory distress; (2) loss of >20% body weight; (3) inability to access food or water for >24 h. Animals meeting these criteria were euthanized immediately by CO_2_ inhalation followed by cervical dislocation.

#### 2.1.3. Sample Size Calculation

Sample size was calculated a priori using G*Power 3.1.9.7 software. The primary endpoint was myocardial infarct size. Based on preliminary data (*n* = 5 per group), the MIRI group showed an infarct size of 45 ± 8%, while the HSYA (20 mg/kg) group showed 28 ± 7%. Using an independent *t*-test with alpha = 0.05 (two-tailed), power = 0.80, and effect size d = 2.12, the minimum sample size required was 6 animals per group. To account for anticipated attrition (estimated at 15–20% based on surgical complexity), we set *n* = 8–12 per group, providing adequate statistical power.

#### 2.1.4. Randomization and Blinding

Animals were randomly allocated to experimental groups using computer-generated random numbers (GraphPad Prism 9.0). Randomization was performed by an independent investigator not involved in surgery or outcome assessment. Group allocation was concealed using coded syringes and animal cages. The surgeon performing LAD ligation was blinded to group assignment. Outcome assessors analyzing ECG, echocardiography, and histology were blinded to group identity until final data analysis.

#### 2.1.5. Exclusion Criteria

Animals were excluded if they met any of the following criteria: (1) ST-segment elevation > 0.1 mV before LAD ligation (suggesting pre-existing cardiac injury); (2) death within 30 min of reperfusion (surgical failure); (3) ventricular fibrillation lasting >5 min without spontaneous conversion; (4) failure to achieve >85% reduction in coronary blood flow after LAD ligation (assessed by laser speckle imaging). Cell samples were excluded if (1) cell viability was <85% before OGD/R induction; (2) mycoplasma contamination was detected; (3) siRNA transfection efficiency was <70%.

### 2.2. Reagents

Hydroxysafflower Yellow A (HSYA, No: BH20968, purity > 98%) was purchased from Shanghai Yuanye Biotechnology Co., Ltd., Shanghai, China; Trimetazidine (Batch No.: 20240421) was purchased from Shandong Sparkjade Biotechnology Co., Ltd., Jinan, China; Autophagy inhibitor 3-methyladenine (3-MA, No: S276713) was purchased from Selleck Chemicals LLC, Shanghai, China; SIRT1 inhibitor EX527 (Lot No: S154106) was purchased from Selleck Chemicals LLC; Creatine kinase-MB (CK-MB) detection kit (Lot No.: JL2024070006) was purchased from Shandong Sparkjade Biotechnology Co.; Cardiac troponin I (cTnI) detection kit (Batch No.: JL2024060001) was purchased from Shandong Sparkjade Biotechnology Co.; Lactate dehydrogenase (LDH) detection kit (Batch No.: JL2024070001) was purchased from Shandong Sparkjade Biotechnology Co.; SIRT1 antibody (Batch No.: ab110304) was purchased from Abcam plc, Shanghai, China; FOXO3 antibody (Batch No: RM0393) was purchased from Suzhou Biodragon Technology Co., Ltd., Suzhou, China; BNIP3 antibody (Batch No.: ab109362) was purchased from Abcam plc; P62 antibody (Batch No.: 29503-1-AP) was purchased from Proteintech Group, Inc., Wuhan, China; Beclin1 antibody (Batch No.: RM4125) was purchased from Suzhou Biodragon Technology Co., Ltd.; LC3 antibody (Batch No.: 35Y4418) was purchased from Affinity Biosciences, Liyang, China; GAPDH antibody (Batch No.: R380626) was purchased from Chengdu ZenBio Biotechnology Co., Ltd., Chengdu, China.

### 2.3. Untargeted Metabolomics

Myocardial tissue samples from the infarct region of mice heart in the sham-operated group and MIRI group were collected, rapidly frozen in liquid nitrogen, and ground into powder. A total of 8 mice per group were included (*n* = 8) to ensure adequate statistical power. Quality control (QC) samples were prepared by pooling equal aliquots of all tissue homogenates and analyzed every 8 injections throughout the run to monitor instrument stability. A pre-cooled extraction solvent (methanol–water, 4:1) was added to the tissue powder, followed by vortex mixing and ultrasonic extraction. The mixture was centrifuged at 12,000× *g* for 15 min at 4 °C, and the supernatant was collected and dried under vacuum. The dried residue was reconstituted with an appropriate solvent and filtered through a 0.22 μm membrane filter. Ultra-performance liquid chromatography–tandem mass spectrometry (UPLC-MS/MS) was used for metabolite detection. The mobile phase consisted of acetonitrile and 0.1% formic acid aqueous solution with gradient elution, and the mass spectrometer was operated in both positive and negative ion modes. Raw data were preprocessed using XCMS software (version 4.10.0) for peak identification, alignment, and quantification. Metabolites were annotated by matching with public databases. Multivariate statistical analyses (principal component analysis, PCA; orthogonal partial least squares–discriminant analysis, OPLS-DA) were performed to screen for differential metabolites, which were further validated by univariate statistics (*t*-test and variable importance in projection [VIP] values). Differential metabolites were subjected to Kyoto Encyclopedia of Genes and Genomes (KEGG) pathway enrichment analysis. Target genes associated with enriched pathways were retrieved, and intersects with HSYA-predicted targets from network pharmacology were identified; the SIRT1-FOXO3-BNIP3 axis was prioritized due to its central role in mitophagy and cardiac homeostasis.

### 2.4. Molecular Docking

The crystal structure of SIRT1 (ID: 4I5I) was retrieved from the Protein Data Bank (PDBID) database, and the small-molecule structure of HSYA (in PDB format) was obtained from the PubChem database (CID: 5281672). Both structures were imported into AutoDock (version 4.2.6) for preprocessing (including removal of water molecules, addition of polar hydrogen atoms, and assignment of partial charges), yielding the corresponding PDBQT files. Grid maps were generated using AutoGrid (version 4.2.9), and molecular docking simulations were subsequently performed with AutoDock to acquire docking results. The docking data were analyzed using AutoDock Tools, and the protein–ligand interactions were visualized with PyMOL software (vision 3.1.8).

### 2.5. Surface Plasmon Resonance

The binding affinity between HSYA and SIRT1 protein was determined using surface plasmon resonance (SPR) on a Biacore T200 instrument (Cytiva, Wilmington, DE, USA). The recombinant SIRT1 protein was immobilized onto a CM5 sensor chip via amine coupling according to the manufacturer’s instructions. HSYA was dissolved in running buffer (10 mM HEPES, 150 mM NaCl, 3 mM EDTA, and 0.05% Tween-20; pH 7.4) and injected at a series of concentrations (4.1 μM, 12.3 μM, 37 μM, 111.1 μM, and 333.3 μM) with a flow rate of 30 μL/min at 25 °C. The sensor surface was regenerated between injections using an appropriate regeneration solution. Equilibrium dissociation constant (KD) values were calculated using the Biacore Evaluation Software (version 6.0) by fitting the response units against analyte concentrations using a 1:1 Langmuir binding model.

### 2.6. Electrocardiography

Mice were fixed in a supine position on the operating table. Following anesthesia with 1.5–2.0% isoflurane delivered via a nose cone (inhalation anesthesia), a stabilization period of 5–10 min was allowed until consistent heart rate and respiratory patterns were achieved. Once hemodynamic parameters were stabilized, ECG signals were recorded using the BL-420 biological signal acquisition system via limb lead II. ECG tracings were obtained before and after drug treatment in each group, and ST-segment deviation was measured for quantitative analysis.

### 2.7. Laser Speckle Blood Flow Imager

Mice were anesthetized via inhalation of isoflurane (induction 3%, maintenance 1.5%) and placed in a left lateral decubitus position. Depilatory cream was evenly applied to the chest skin for hair removal, and ultrasonic coupling gel was spread on the depilated area. A high-resolution ultrasound system (Vevo 2100, VisualSonics, Toronto, ON, Canada) equipped with a 30 MHz linear array transducer (MS400) was used. Two-dimensional parasternal long-axis views were obtained at the level of the papillary muscles. M-mode images were recorded from the parasternal short-axis view at the mid-papillary muscle level. An ultrasonic probe was used to locate the heart, and echocardiographic data were collected. Left ventricular end-diastolic diameter (LVEDD) and left ventricular end-systolic diameter (LVESD) were measured from M-mode tracings, and left ventricular ejection fraction (LVEF) and fractional shortening (LVFS) were calculated using the Teichholz formula. LVEF and LVFS were calculated using built-in software to evaluate left ventricular systolic function.

### 2.8. Echocardiography

Mice were anesthetized via inhalation. Depilatory cream was evenly applied to the chest skin for hair removal, and ultrasonic coupling gel was spread on the depilated area. An ultrasonic probe was used to locate the heart, and echocardiographic data were collected. LVEF and LVFS were calculated using built-in software to evaluate left ventricular systolic function.

### 2.9. ELISA

Blood samples and myocardial tissue homogenates, AC16 cells were centrifuged to collect the supernatants. The levels of cardiac troponin I (cTnI), creatine kinase-MB (CK-MB), and lactate dehydrogenase (LDH) in tissue supernatants and serum were detected using commercial ELISA kits according to the manufacturer’s instructions.

### 2.10. TTC Staining

At 2 h after reperfusion, blood samples were collected from the cardiac chamber of mice in each group immediately. Plasma was separated by centrifugation, and the supernatant was stored at −80 °C for subsequent analysis. Mice were sacrificed by cervical dislocation after blood collection, and hearts were excised under sterile conditions. The hearts were frozen at 4 °C, fixed, and then sectioned transversely. Ischemic myocardium was stained red, while infarcted myocardium appeared pale white. After fixation in 4% paraformaldehyde for 24 h, the heart sections were photographed with a digital camera. The infarct size was quantified using ImageJ software (1.54t), and the infarct volume was expressed as a percentage of the total left ventricular volume.

### 2.11. Hematoxylin-Eosin (HE) Staining

Left ventricular myocardial tissues from mice fixed in 10% formalin were washed, followed by routine dehydration, clearing, embedding in paraffin, and serial sectioning at 4 μm. The paraffin sections were deparaffinized with xylene and rehydrated through a graded ethanol series. HE staining was performed according to standard protocols, and the sections were finally mounted with neutral balsam. Pathological changes and cardiomyocyte apoptosis were observed under a light microscope. Five random fields were selected from each section for observation and photography to analyze morphological characteristics.

### 2.12. Transmission Electron Microscopy (TEM)

Infarcted myocardial tissue samples (approximately 1 mm^3^) were rapidly excised and immersed in 2.5% glutaraldehyde solution in 0.1 M phosphate buffer (pH 7.4) for at least 4 h at 4 °C for primary fixation, followed by post-fixation in 1% osmium tetroxide solution in 0.1 M phosphate buffer for 2 h at 4 °C. Subsequently, the tissues were dehydrated through a graded acetone series (30%, 50%, 70%, 80%, 90%, and 100%; 15 min each) and stained with uranyl acetate (2% in 70% acetone) for 30 min during dehydration. The samples were then infiltrated with epoxy resin (EPON 812) and polymerized at 60 °C for 48 h. sectioned into ultra-thin slices (50–80 nm) using an ultramicrotome, counterstained with lead citrate (0.3% in 0.1 M NaOH) for 5 min, and mounted on copper grids. Ultrastructural changes of cardiomyocytes and mitochondria were observed under a TEM at 80 kV accelerating voltage.

### 2.13. Mitochondrial Membrane Potential

Cardiomyocytes isolated from ischemic myocardial tissue and AC16 Cells were transferred to centrifuge tubes and centrifuged at 1000× *g* for 5 min. The supernatant was discarded, and the cells were gently resuspended in phosphate-buffered saline (PBS) and counted. Approximately 5 × 10^4^ to 1 × 10^5^ cells were collected by centrifugation at 1000× *g* for 5 min. After discarding the supernatant, 195 μL of JC-1 staining buffer was added to resuspend the cells, followed by the addition of 5 μL of JC-1 probe solution. The mixture was gently vortexed and incubated at 37 °C for 20 min in the dark. A negative control group (without JC-1 probe) was set up simultaneously. Following incubation, cells were washed twice with pre-cooled PBS and resuspended in 300 μL of PBS for flow cytometric analysis. Samples were analyzed on a BD FACSCanto II flow cytometer (BD Biosciences, Franklin Lakes, NJ, USA) equipped with 488 nm and 561 nm lasers. Data acquisition was performed using BD FACSDiva software (version 8.0.2), with a minimum of 10,000 events collected per sample. The sorting strategy was as follows: (i) cells were first gated on forward scatter (FSC-A) versus side scatter (SSC-A) to exclude debris and select the main cell population; (ii) doublets were excluded using FSC-A versus FSC-H gating; (iii) JC-1 aggregates (high MMP) emitting red fluorescence were detected in the PE channel (excitation 561 nm; emission 585 ± 15 nm); (iv) JC-1 monomers (low MMP) emitting green fluorescence were detected in the FITC channel (excitation 488 nm; emission 530 ± 15 nm). The negative control (without JC-1) was used to set the baseline fluorescence threshold for both channels. The MMP level was quantified as the ratio of red to green fluorescence intensity, and data were analyzed using FlowJo software (version 10.8.1, BD Biosciences). JC-1 aggregates (indicating high MMP) emitted red fluorescence (detected in the PE channel) and JC-1 monomers (indicating low MMP) emitted green fluorescence (detected in the FITC channel). The MMP level was expressed as the percentage of JC-1 aggregates in vivo and the MMP level was quantified as the ratio of red to green fluorescence intensity in vitro.

### 2.14. Immunofluorescence Staining

Paraffin-embedded myocardial tissue sections and AC16 Cells were deparaffinized, rehydrated, and subjected to antigen retrieval by boiling in citrate buffer. After blocking with 5% bovine serum albumin (BSA) for 1 h at room temperature, the sections were incubated with primary antibodies against SIRT1 (1:500), FOXO3 (1:500), BNIP3 (1:500), and LC3 (1:500) overnight at 4 °C. The next day, the sections were washed with TBST and incubated with fluorescently labeled secondary antibodies (1:1000) for 1 h at room temperature in the dark. Nuclei were counterstained with 4′,6-diamidino-2-phenylindole (DAPI) for 5 min. The expression levels and localization of target proteins were observed and imaged using a confocal laser scanning microscope (CLSM Leica Microsystems, Wetzlar, Germany).

### 2.15. Western Blot

Myocardial tissue samples were lysed in RIPA lysis buffer containing 1% phenylmethylsulfonyl fluoride (PMSF), 1% sodium deoxycholate, and protease inhibitor cocktail. The protein concentration was determined using a BCA protein assay kit according to the manufacturer’s instructions. Equal amounts of protein (30 μg per lane) were mixed with loading buffer and denatured by boiling at 95 °C for 5 min. Sodium dodecyl sulfate-polyacrylamide gel electrophoresis (SDS-PAGE) was performed on 10–15% separating gels with 5% stacking gels, and the proteins were transferred to polyvinylidene fluoride (PVDF) membranes using a wet transfer system (Bio-Rad, Hercules, CA, USA) at 100 V for 90 min at 4 °C in transfer buffer (25 mM Tris, 192 mM glycine, and 20% methanol). The membranes were blocked with 5% non-fat milk in TBST for 1 h at room temperature, then incubated with primary antibodies against SIRT1 (1:1000), FOXO3 (1:1000), BNIP3 (1:1000), LC3 (1:500), Beclin-1 (1:1000), P62 (1:1000), and GAPDH (1:10,000, internal control) overnight at 4 °C. After washing with TBST (three times, 10 min each), the membranes were incubated with horseradish peroxidase (HRP)-conjugated secondary antibodies (1:10,000) for 2 h at room temperature. After washing three times with TBST (10 min each), protein bands were visualized using enhanced chemiluminescence (ECL) reagent, and the relative protein expression levels were quantified by densitometric analysis using ImageJ software. The results were expressed as the ratio of the target protein band intensity to the GAPDH band intensity.

### 2.16. Oxygen-Glucose Deprivation/Reoxygenation (OGD/R) Model in AC16 Cells and Experimental Grouping

To simulate myocardial ischemia-reperfusion injury in vitro, AC16 cardiomyocytes were subjected to OGD/R treatment. Briefly, cells were cultured in glucose-free DMEM medium under hypoxic conditions (5% CO_2_, 1% O_2_, and 94% N_2_) for 4 h, followed by reoxygenation in complete DMEM medium containing 10% fetal bovine serum (FBS) under normoxic conditions (5% CO_2_; 95% air) for 8 h. For investigating the cardioprotective effect of HSYA, cells were divided into the following groups: control group (no OGD/R treatment), OGD/R group, HSYA groups (OGD/R + 1.25, 5, and 20 μmol/L), and positive control group (OGD/R + 100 μmol/L Trimetazidine, Tri). To explore the role of SIRT1 in mitophagy, additional groups were set up: control group, OGD/R group, HSYA group (OGD/R + 20 μmol/L), negative control (NC) group (OGD/R + non-targeting siRNA), si-SIRT1 group (OGD/R + SIRT1-targeting siRNA), and si-SIRT1 + HSYA group (OGD/R + SIRT1-targeting siRNA + 20 μmol/L HSYA).

### 2.17. Cell Viability

AC16 cells in the logarithmic growth phase were seeded into 96-well plates at a density of 2 × 10^4^ cells/well. After cell adhesion, cells were treated according to experimental grouping. Ten microliters of CCK-8 solution was added to each well, and the plates were incubated at 37 °C for 1 h. The absorbance at 450 nm was measured using a microplate reader. All experiments were performed in triplicate, and results were expressed as mean ± standard deviation (SD). Cell viability was calculated using the following formula: cell viability (%) = [(A_treaten_ − A_βlank_)/(A_ontrol_ − A_βlank_)] × 100%, where A_treaten_ = absorbance of treated groups, A_ontrol_ = absorbance of control group, and A_βlank_ = absorbance of blank well (medium + CCK-8 solution without cells).

### 2.18. SIRT1 Silencing by siRNA Transfection in AC16 Cells

SIRT1-targeting small interfering RNA (siRNA) and non-targeting control siRNA were purchased from Shandong Weizhen Biotechnology Co., Ltd., Jinan, China. AC16 cells were seeded into 6-well plates and transfected with siRNA using a commercial transfection reagent, following the manufacturer’s protocol. Briefly, 100 μL of transfection mixture (siRNA + transfection reagent diluted in serum-free medium) was added to each well containing 100 μL of serum-free medium. The siRNA sequences used were as follows:

si-SIRT1-1: GCCATGTTTGATATTGAGTAT

si-SIRT1-2: AGTGAGACCAGTAGCACTAAT

si-SIRT1-3: GAGGGTAATCAATACCTGTTT

At 48 h post transfection, the efficiency of SIRT1 silencing was verified by Western blot analysis (Section 2.14) before subsequent OGD/R treatment and HSYA intervention.

### 2.19. Lysosomal Inhibitor Intervention

To verify the regulatory effect of HSYA on autophagic activity and distinguish the promotion of autophagosome formation from lysosomal degradation blockade, bafilomycin A1 (BafA1) was used to inhibit lysosomal degradation function in AC16 cardiomyocytes. AC16 cells were seeded in culture plates and subjected to oxygen-glucose deprivation/reoxygenation (OGD/R) injury as previously described. For lysosomal inhibition treatment, cells were incubated with 20 nM BafA1 (MedChemExpress, Monmouth Junction, NJ, USA) for 4 h before cell collection according to the classic experimental protocol reported in published studies [24]. Cells were divided into three groups: OGD/R group, OGD/R + BafA1 group, and OGD/R + BafA1 + HSYA treatment group. After corresponding interventions, cells were washed twice with pre-cooled phosphate buffered saline (PBS), and total cellular protein was extracted using RIPA lysis buffer supplemented with protease inhibitor cocktail. The protein concentration of each sample was quantified by BCA protein assay kit, and equal amounts of protein samples were denatured at 100 °C for 10 min. Subsequent experimental procedures were performed in accordance with those described in Section 2.15.

### 2.20. Transmission Electron Microscopy

AC16 cells were seeded in culture dishes and cultured until the target time point. After fixation with 2.5% glutaraldehyde at 4 °C for 4 h, cells were post-fixed with 1% osmium tetroxide for 1 h. Subsequently, the cells were dehydrated through a graded ethanol series (30%, 50%, 70%, 90%, and 100%) and embedded in epoxy resin. Ultra-thin sections (50–80 nm) were cut using an ultramicrotome, stained with uranyl acetate and lead citrate, and mounted on copper grids. Mitochondrial morphology and cellular ultrastructural changes were observed under a TEM, and images were captured for analysis.

### 2.21. Pull-Down

Bait proteins (GST-tagged SIRT1/FOXO3/BNIP3) and prey proteins (His-tagged FOXO3/BNIP3/LC3B) were expressed and purified from prokaryotic cells. GST affinity resin was washed three times with pre-cooled binding buffer and resuspended to form a 50% resin slurry. GST-tagged bait proteins were added to the resin, and the mixture was incubated with shaking at 4 °C for 1–2 h to form GST–bait–resin complexes. Purified prey proteins or total cell lysates (containing endogenous prey proteins) were then added, followed by incubation with gentle shaking at 4 °C for 2–4 h. A negative control group (GST protein alone bound to resin) was set up to exclude non-specific binding. The resin complexes were washed 4–5 times with washing buffer (containing low-concentration NaCl) to remove unbound proteins. Bound proteins were eluted with GST elution buffer (50 mM Tris-HCl and 10 mM reduced glutathione; pH 8.0) by incubation at room temperature for 10–15 min. The eluates were collected, subjected to SDS-PAGE electrophoresis, and analyzed by Western blot using antibodies against the prey protein or tags (His/GST) to verify the interaction.

### 2.22. Statistical Analysis

All experimental data were expressed as mean ± standard deviation (SD) and analyzed using SPSS 26.0 software (IBM Corp., Armonk, NY, USA). Prior to parametric testing, the normality of data was assessed by the Shapiro–Wilk test, and homogeneity of variances was verified using Levene’s test. For comparisons of means among multiple groups, one-way analysis of variance (ANOVA) was performed followed by Tukey’s post-hoc test. For HSYA concentration screening experiments, two-way ANOVA was used to evaluate the interaction between factors (e.g., HSYA concentration and OGD/R treatment), and Tukey’s post-hoc test was subsequently applied to analyze pairwise group differences. Statistical significance was defined as *p* < 0.05. It is important to note that ANOVA-based analyses establish statistical associations among variables and do not, by themselves, infer causality. To strengthen causal interpretation, this study employed complementary functional perturbation strategies: (i) pharmacological inhibition of SIRT1 using EX527 in vivo and (ii) siRNA-mediated genetic silencing of SIRT1 in vitro, both followed by HSYA rescue interventions. The rationale for causal inference is based on the following logic: if HSYA exerts its protective effects through the SIRT1-FOXO3-BNIP3 pathway, then blocking or silencing SIRT1 should attenuate or abolish HSYA’s effects, and this attenuation should be partially reversible upon HSYA administration (rescue experiment). The consistency of results across both pharmacological and genetic loss-of-function approaches, combined with the rescue paradigm, provides convergent evidence supporting a causal role of SIRT1-FOXO3-BNIP3 axis activation in HSYA-mediated cardio protection.

## 3. Results

### 3.1. Screening of Drug Action Targets by Network Pharmacology and Metabolomics

**Figure 1:**
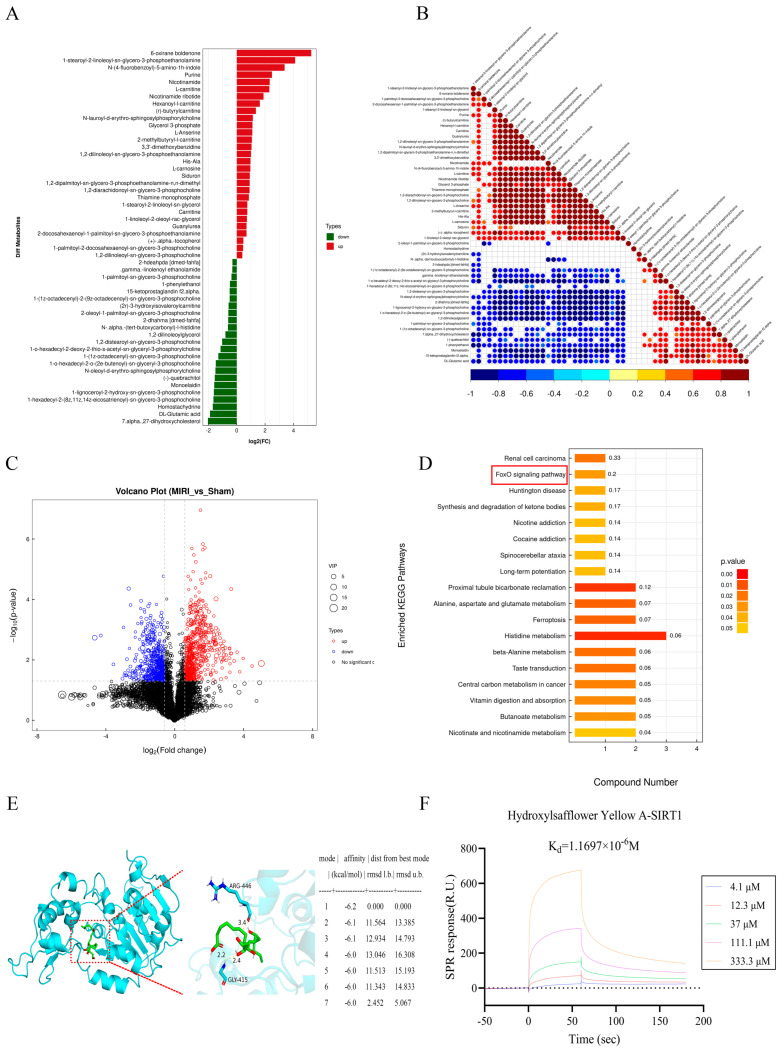
Analysis of differential metabolites and screening of therapeutic targets. (**A**,**B**) Differential metabolites between the sham and MIRI groups. (**C**) Volcano plot showing differential metabolites between the sham and MIRI groups, where red and blue dots represent significantly differential metabolites (*p* < 0.05). (**D**) KEGG enrichment analysis (The red frame is the enriched pathway). (**E**) Molecular docking between HSYA and SIRT1. (**F**) Surface plasmon resonance.

Untargeted metabolomics analysis was performed on the sham and MIRI groups to screen for differential metabolites (Figure 1A,B), followed by enrichment analysis of these differential metabolites using a volcano plot (Figure 1C). KEGG pathway enrichment analysis of the differential metabolites (Figure 1D). Molecular docking results revealed a strong binding affinity between HSYA and SIRT1 (Figure 1E). As depicted in the sensorgram, HSYA exhibited a pronounced concentration-dependent binding profile to immobilized SIRT1 across the tested concentrations (4.1, 12.3, 37, 111.1, and 333.3 μM). During the association phase (0–50 s), the SPR response units (RU) rose rapidly, with higher HSYA concentrations correlating with faster association rates and greater maximal binding signals. Upon entering the dissociation phase (after 50 s), the response signals gradually declined, and the residual RU at equilibrium was positively correlated with the initial HSYA concentration. These characteristics indicate a concentration-dependent stability of the HSYA–SIRT1 complex, confirming a direct and specific interaction between HSYA and SIRT1 (Figure 1F).

### 3.2. HSYA Reduces Abnormal ST-Segment Elevation and Improves Myocardial Blood Perfusion and Cardiac Function After MIRI

**Figure 2:**
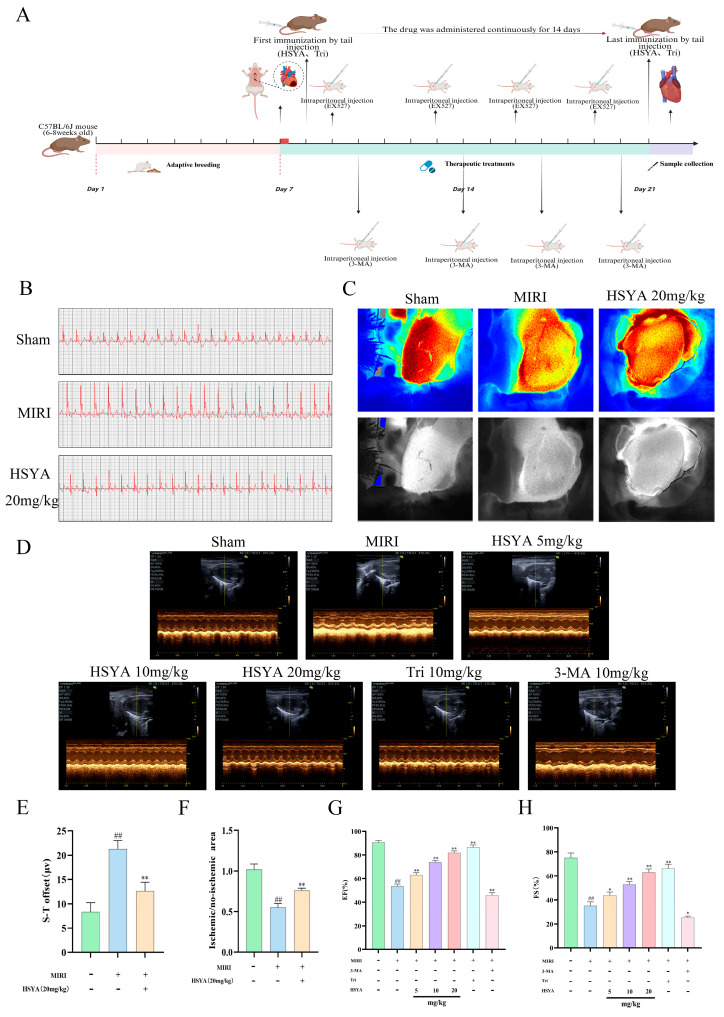
HSYA attenuates abnormal ST-segment elevation and improves myocardial perfusion and cardiac output in mice after MIRI. (**A**) Drug administration schedule in mice. (**B**) Electrocardiograms (ECGs) of mice (*n* = 3). (**C**) Laser speckle blood flow imager of the ischemic region in mice (*n* = 3). (**D**) Echocardiograms of mice (*n* = 3). (**E**) Statistical Analysis of Mouse ST-segment Deviation. (**F**) Statistical analysis of murine laser speckle perfusion imaging. (**G**) Left ventricular ejection fraction (LVEF). (**H**) Left ventricular fractional shortening (LVFS). ^##^ *p* < 0.01 vs. sham group; * *p* < 0.05, ** *p* < 0.01 vs. model group.

The timeline of drug administration in C57BL/6J mice is shown (Figure 2A). Electrocardiographic monitoring was performed after MIRI induction. Compared with the sham group, the MIRI group exhibited a significant increase in ST-segment deviation (*p* < 0.01), while administration of HSYA (all doses) and Tri significantly ameliorated abnormal ST-segment elevation (*p* < 0.05 or *p* < 0.01 vs. MIRI group) (Figure 2B,C). Laser speckle blood flow imager results demonstrated that, in contrast to the sham group, HSYA treatment significantly enhanced blood perfusion in the ischemic region of the left ventricle (*p* < 0.01 vs. sham group; *p* < 0.05 or *p* < 0.01 vs. MIRI group) (Figure 2D,E). Additionally, echocardiography was used to evaluate cardiac function indices after MIRI. LVEF was markedly decreased in the MIRI group compared with the sham group (*p* < 0.01), whereas HSYA (all doses) and Tri treatment significantly improved LVEF (*p* < 0.05 or *p* < 0.01 vs. MIRI group). Similarly, LVFS was significantly reduced in the MIRI group (*p* < 0.01 vs. sham group), and HSYA (all doses) and Tri effectively restored LVFS (*p* < 0.05 or *p* < 0.01 vs. MIRI group) (Figure 2F–H).

### 3.3. HSYA Reduces the Release of Myocardial Injury Markers and Attenuates Pathological Damage in Ischemic Myocardium of Mice

**Figure 3:**
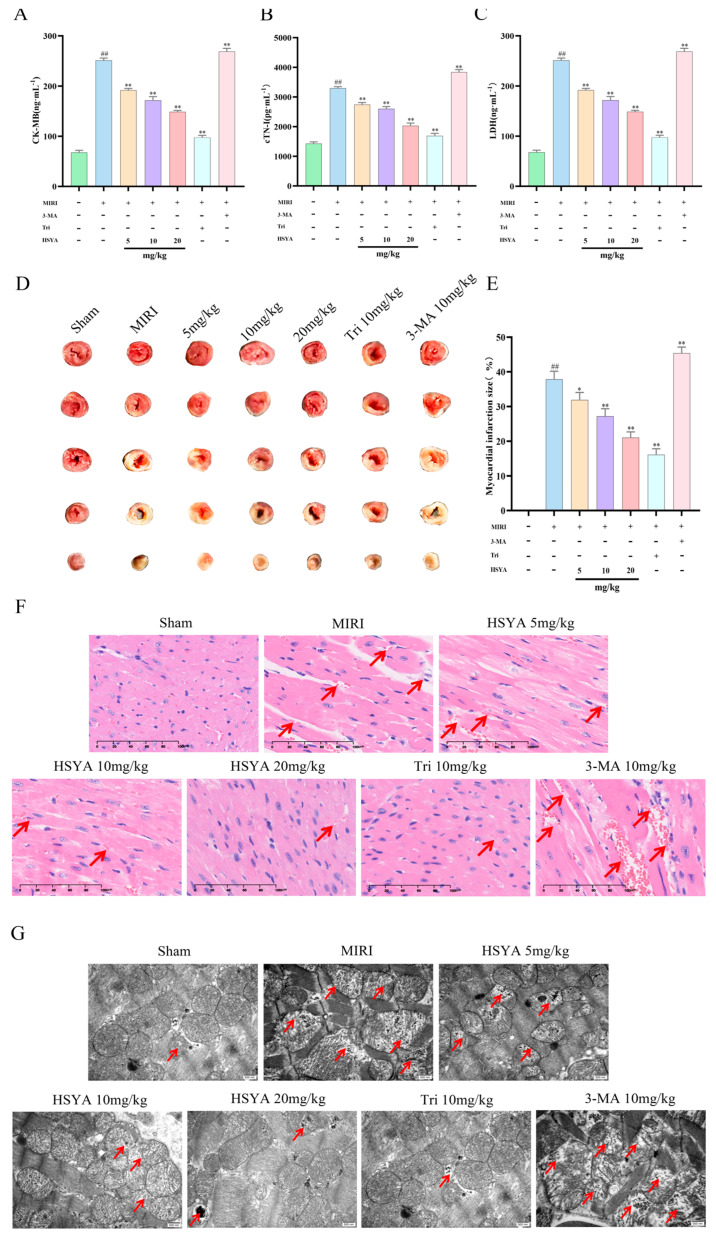
HSYA reduces the release of myocardial injury markers, ameliorates pathological damage of ischemic myocardium, and protects myocardial tissue in mice. (**A**) Creatine kinase isoenzyme MB (CK-MB) (*n* = 6). (**B**) Cardiac troponin I (cTnI) (*n* = 6). (**C**) Lactate dehydrogenase (LDH) (*n* = 6). (**D**,**E**) TTC staining analysis of myocardial infarct size in mice (*n* = 3). (**F**) Hematoxylin-eosin (HE) staining of mouse myocardial tissue (*n* = 6); red arrows indicate areas of inflammatory cell infiltration. (**G**) Transmission electron microscopy (TEM) of mouse ischemic myocardium (25,000×); red arrows indicate damaged mitochondria with disrupted cristae and compromised membrane integrity. ^##^ *p* < 0.01 vs. sham group; * *p* < 0.05, ** *p* < 0.01 vs. model group.

ELISA results showed that compared with the sham group, the levels of creatine kinase-MB (CK-MB), lactate dehydrogenase (LDH), and cardiac troponin I (cTnI) in tissue supernatants were significantly increased in the MIRI group (all *p* < 0.01). After administration, HSYA (all doses) and Tri effectively decreased the levels of these injury markers (all *p* < 0.01 vs. MIRI group), indicating that HSYA can alleviate myocardial injury in mice with MIRI (Figure 3A–C). TTC staining was performed on myocardial tissue from the ischemic region of each group. Compared with the MIRI group, HSYA (all doses) and Tri groups significantly reduced the infarct size in the ischemic myocardium (*p* < 0.05 or *p* < 0.01 vs. MIRI group), thereby attenuating MIRI. In contrast, the infarct size was further increased in the 3-MA (autophagy inhibitor) group compared with the MIRI group (*p* < 0.05), suggesting that inhibition of autophagy exacerbated myocardial ischemia-reperfusion injury (Figure 3D,E). HE staining of the left ventricular infarct region revealed severe pathological damage in the MIRI group, including disorganized myocardial fibers, interstitial edema, neutrophil infiltration, and obvious myofilament disruption. Compared with the MIRI group, HSYA (all doses) and Tri groups showed significantly mitigated myocardial tissue damage (all *p* < 0.01), characterized by mildly disorganized myocardial fibers, slight myofilament disruption, minimal neutrophil infiltration, and mild interstitial edema (Figure 3F). TEM observations demonstrated that mitochondria in the MIRI group were markedly swollen, with disrupted cristae and impaired membrane integrity. After HSYA or Tri treatment, mitochondrial swelling and cristae disruption were significantly ameliorated (*p* < 0.01 vs. MIRI group). In contrast, 3-MA treatment further aggravated mitochondrial damage, with blurred mitochondrial boundaries and complete loss of cristae (*p* < 0.05 vs. MIRI group), indicating that inhibition of mitophagy exacerbates myocardial injury (Figure 3G). Collectively, these results demonstrate that HSYA alleviates MIRI in mice by reducing myocardial injury markers, decreasing infarct size, and improving pathological damage in ischemic myocardium.

### 3.4. HSYA Attenuates Mitochondrial Damage and Ameliorates Myocardial Injury After MIRI in Mice

**Figure 4:**
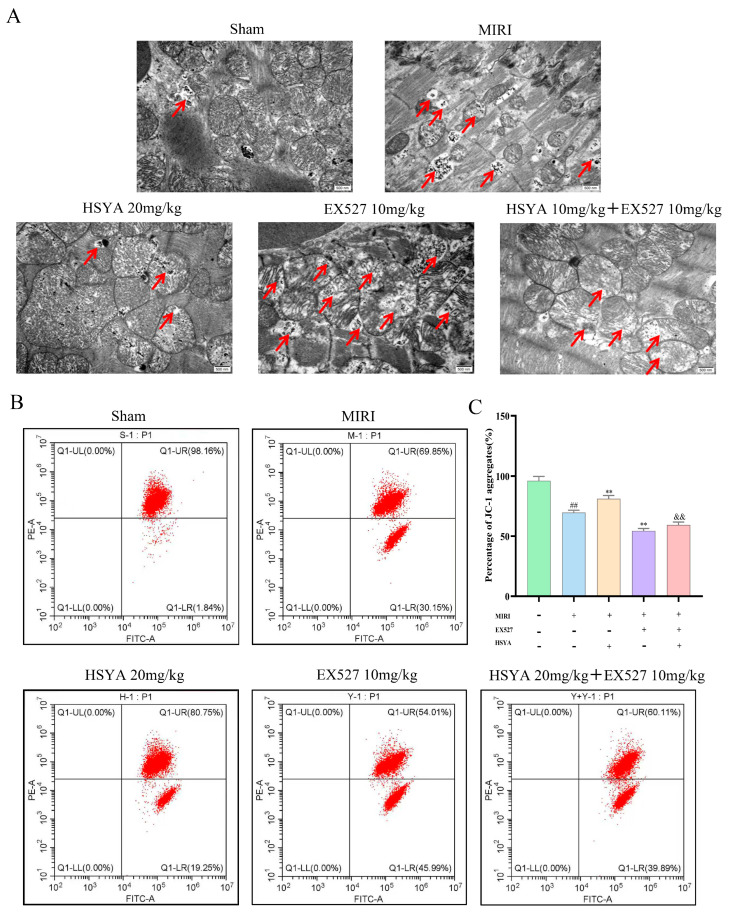
HSYA improves mitochondrial morphology and function, reduces mitochondrial membrane potential (MMP), and attenuates myocardial tissue injury. (**A**) Transmission electron microscopy (TEM) images showing mitochondrial morphology and autophagosomes (25,000×; *n* = 3); red arrows indicate damaged mitochondria with disrupted cristae and compromised membrane integrity. (**B**) Flow cytometry detection (*n* = 3). (**C**) Quantitative analysis of mitochondrial membrane potential (MMP). ^##^ *p* < 0.01 vs. sham group; ** *p* < 0.01 vs. model group; ^&&^ *p* < 0.01 vs. HSYA 20 mg/kg group.

Transmission electron microscopy (TEM) was used to observe myocardial tissue in the ischemic region of mice. Compared with the sham group, mitochondria in the MIRI group were markedly swollen, deformed, and disorganized with disrupted cristae and compromised membrane integrity. After administration of HSYA (20 mg/kg), mitochondrial swelling, deformation, and cristae disruption were significantly ameliorated; mitochondria were closely arranged with relatively intact membrane structures. Treatment with the SIRT1 inhibitor EX527 resulted in complete disruption of the mitochondrial membrane structure, while combined treatment with EX527 and HSYA (20 mg/kg) significantly improved mitochondrial morphology compared with EX527 alone (Figure 4A). Flow cytometry analysis showed that the mitochondrial membrane potential (MMP) was significantly decreased in the MIRI group compared with the sham group (*p* < 0.01), indicating severe mitochondrial depolarization after MIRI. HSYA (20 mg/kg) treatment significantly increased MMP (*p* < 0.01 vs. MIRI group) and restoration of mitochondrial membrane potential, thereby alleviating mitochondrial dysfunction. Inhibition of SIRT1 expression led to a further decrease in MMP compared with the MIRI group (*p* < 0.01), suggesting aggravated myocardial damage. Notably, combined administration of HSYA and EX527 significantly increased MMP compared with EX527 alone (*p* < 0.01), thereby partially reversing EX527-induced mitochondrial damage (Figure 4B,C).

### 3.5. HSYA Activates the SIRT1-FOXO3-BNIP3 Signaling Pathway to Promote Mitophagy

**Figure 5:**
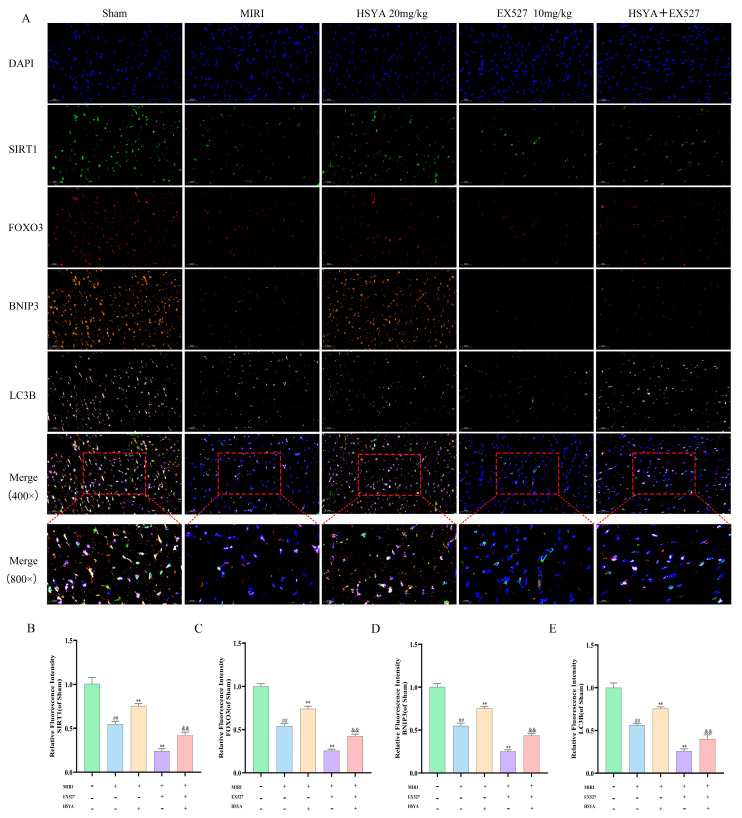
HSYA activates the SIRT1-FOXO3-BNIP3 signaling pathway and enhances the fluorescence intensity of SIRT1, FOXO3, BNIP3, and LC3B. (**A**) Immunofluorescence staining (400×) showing the fluorescence intensity of SIRT1, FOXO3, BNIP3, and LC3B (*n* = 3). (**B**) Statistical analysis of SIRT1 fluorescence intensity. (**C**) Statistical analysis of FOXO3 fluorescence intensity. (**D**) Statistical analysis of BNIP3 fluorescence intensity. (**E**) Statistical analysis of LC3B fluorescence intensity. ^##^ *p* < 0.01 vs. sham group; ** *p* < 0.01 vs. model group; ^&&^ *p* < 0.01 vs. HSYA 20 mg/kg group.

Immunofluorescence quadruple staining results showed that compared with the sham group (Figure 5A), the relative fluorescence intensities of SIRT1, FOXO3, BNIP3, and LC3B were significantly decreased in the MIRI group (all *p* < 0.01). After HSYA (20 mg/kg) administration, the relative fluorescence intensities of these proteins were significantly increased (all *p* < 0.01 vs. MIRI group), indicating activation of the SIRT1-FOXO3-BNIP3 signaling pathway. Further inhibition of SIRT1 expression with EX527 on the basis of MIRI led to a further decrease in the fluorescence intensities of SIRT1, FOXO3, BNIP3, and LC3B compared with the MIRI group (all *p* < 0.01). To verify whether HSYA could reverse the inhibitory effect of EX527, mice were treated with HSYA (20 mg/kg) in combination with EX527. The results demonstrated that the relative fluorescence intensities of SIRT1, FOXO3, BNIP3, and LC3B were significantly higher in the combined treatment group than in the EX527 alone group (all *p* < 0.01), indicating that HSYA partially reversed EX527-mediated suppression of this signaling cascade (Figure 5B–E; Figure S1).

### 3.6. Effects of HSYA on the Expression of SIRT1-FOXO3-BNIP3 Pathway Proteins and Autophagy-Related Proteins

**Figure 6:**
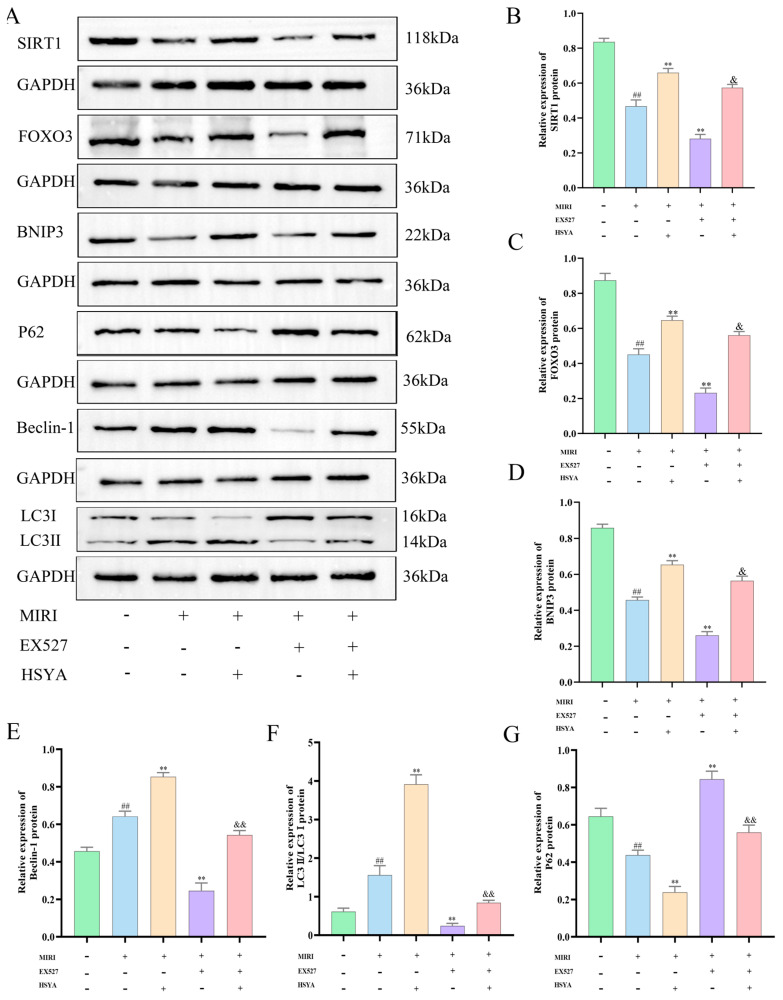
HSYA upregulates the expression of pathway-related proteins and autophagy-related proteins. (**A**) Western blot (WB) analysis of the protein expression of SIRT1, FOXO3, BNIP3, P62, Beclin-1, and LC3I/LC3II (*n* = 3). (**B**) Densitometric analysis of SIRT1 protein bands. (**C**) Densitometric analysis of FOXO3 protein bands. (**D**) Densitometric analysis of BNIP3 protein bands. (**E**) Densitometric analysis of Beclin-1 protein bands. (**F**) Densitometric analysis of LC3II/I protein bands. (**G**) Densitometric analysis of P62 protein bands. ^##^ *p* < 0.01 vs. sham group; ** *p* < 0.01 vs. model group; ^&^ *p* < 0.05, ^&&^ *p* < 0.01 vs. HSYA 20 mg/kg group.

Western blot (WB) results showed that compared with the sham group (Figure 6A), the expression levels of SIRT1, FOXO3, BNIP3, and LC3B (pathway-related proteins) were significantly decreased in the MIRI group (all *p* < 0.01). In contrast, HSYA (20 mg/kg) administration significantly upregulated the expression of these proteins compared with the MIRI group (all *p* < 0.01). On the basis of the MIRI model, treatment with EX527 (SIRT1 inhibitor) further reduced the expression of SIRT1, FOXO3, BNIP3, and LC3B (all *p* < 0.01 vs. MIRI group), while subsequent HSYA administration significantly reversed the EX527-induced downregulation of these proteins (all *p* < 0.01 vs. EX527 group)—consistent with the immunofluorescence findings (Figure 6B–D). Meanwhile, the expression levels of autophagy-related proteins Beclin-1 and the LC3II/I ratio were significantly increased (both *p* < 0.01 vs. MIRI group), and P62 expression was decreased (*p* < 0.01 vs. MIRI group), indicating enhanced autophagic activity (Figure 6E,F). Compared with the MIRI group, the EX527 group exhibited decreased Beclin-1 and LC3II/I expression (both *p* < 0.01), increased P62 expression (*p* < 0.01), and reduced autophagic activity (Figure 6G). Collectively, these results demonstrate that HSYA protects against myocardial ischemia-reperfusion injury by enhancing mitophagy through the SIRT1-FOXO3-BNIP3-LC3B signaling axis.

### 3.7. HSYA Reduces the Release of Myocardial Injury Markers in AC16 Cells

**Figure 7:**
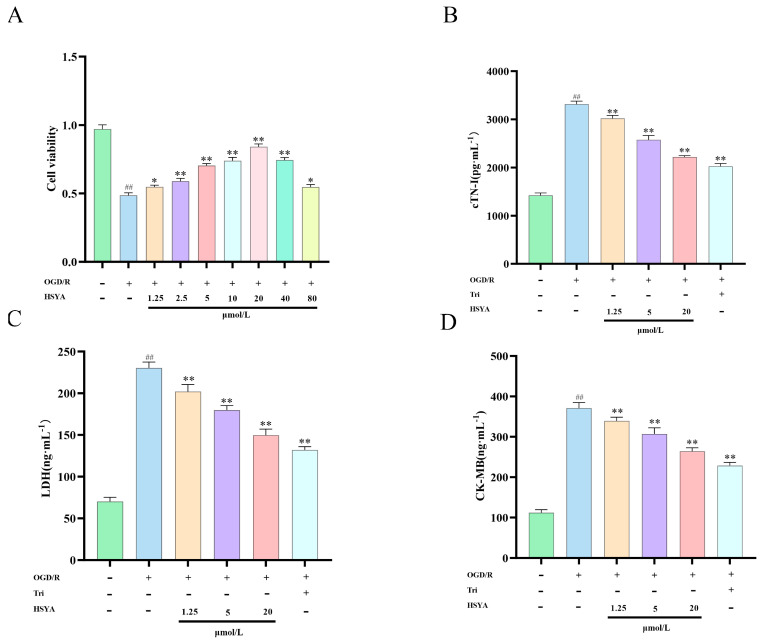
HSYA reduces the release of LDH, cTnI, and CK-MB; attenuates OGD/R-induced injury; and protects AC16 cells. (**A**) CCK-8 assay for cell viability screening (*n* = 6). (**B**) Cardiac troponin I (cTnI) (*n* = 6). (**C**) Lactate dehydrogenase (LDH) (*n* = 6). (**D**) Creatine kinase isoenzyme MB (CK-MB) (*n* = 6). ^##^ *p* < 0.01 vs. control group; * *p* < 0.05, ** *p* < 0.01 vs. OGD/R group.

The optimal concentration of HSYA for AC16 cells was screened using the CCK-8 assay (Figure 7A). Cell viability analysis showed that HSYA at concentrations of 1.25, 5, and 20 μmol/L significantly increased cell survival compared with the OGD/R group (all *p* < 0.01), with 20 μmol/L exhibiting the most pronounced protective effect; this concentration was therefore selected as the optimal dose for subsequent experiments. ELISA results showed that the release of myocardial injury-related markers (LDH, cTnI, and CK-MB) in cell supernatants was significantly increased in the OGD/R group compared with the control group (all *p* < 0.01). After HSYA treatment, the levels of LDH, cTnI, and CK-MB were significantly decreased (all *p* < 0.01 vs. OGD/R group), indicating that HSYA exerts a concentration-dependent protective effect against OGD/R-induced injury in AC16 cells (Figure 7B–D).

### 3.8. HSYA Alleviates Mitochondrial Damage and Improves Mitochondrial Function in AC16 Cells

**Figure 8:**
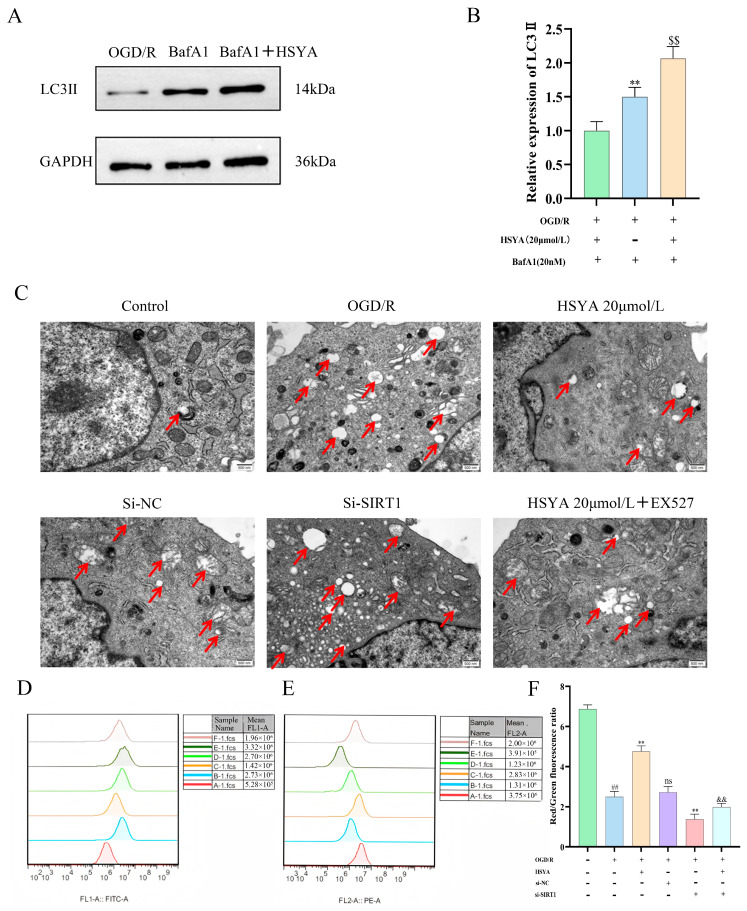
HSYA alleviates mitochondrial injury, mitigates mitochondrial dysfunction, and attenuates OGD/R-induced injury in AC16 cells. (**A**) Lysosomal inhibition intervention. (**B**) Densitometric analysis of protein bands (*n* = 3). (**C**) Transmission electron microscopy (TEM) images of AC16 cells (25,000×; *n* = 3), red arrows indicate damaged mitochondria with disrupted cristae and compromised membrane integrity. (**D**–**F**) Flow cytometry analysis (*n* = 3). ^##^ *p* < 0.01 vs. control group; ** *p* < 0.01 vs. OGD/R group; ^$$^ *p* < 0.01 vs. BafA1 group; ^&&^ *p* < 0.01 vs. HSYA 20 μmol/L group; ns = not significant.

To investigate whether HSYA alleviates OGD/R-induced injury in AC16 cardiomyocytes by promoting mitophagy, LC3II expression was detected by Western blot. Compared with the OGD/R group, LC3II expression was significantly increased after treatment with the lysosomal inhibitor BafA1 (*p* < 0.01), indicating that LC3II could not be rapidly degraded in cells when lysosomal function was inhibited. Notably, LC3II expression was further elevated, and its accumulation was more pronounced after additional treatment with HSYA compared with the BafA1 group (*p* < 0.01), suggesting that HSYA promotes mitophagy and increases mitochondrial autophagic activity in OGD/R-injured AC16 cardiomyocytes. To investigate the role of mitophagy in AC16 cells, adenovirus-mediated SIRT1 silencing was employed to knock down SIRT1 expression. TEM observations revealed that compared with the OGD/R group (model group), HSYA (20 μmol/L) treatment significantly attenuated mitochondrial membrane vacuolization, preserved relatively intact mitochondrial membrane structure, and maintained visible mitochondrial cristae. In contrast, SIRT1 silencing (si-SIRT1 group) resulted in severe mitochondrial vacuolization, complete structural disruption, and loss of cristae, indicating exacerbated mitochondrial damage (Figure 8A). Mitochondrial membrane potential (MMP) analysis revealed that MMP was markedly reduced in the OGD/R group relative to the control group (*p* < 0.01). Treatment with 20 μmol/L HSYA effectively reversed the OGD/R-induced downregulation of MMP (*p* < 0.01) and maintained mitochondrial stability. SIRT1 silencing led to a further decline in MMP compared with the OGD/R group (*p* < 0.01), which implied that suppressed mitophagy worsened mitochondrial structural and functional disorders. Moreover, co-administration of HSYA and si-SIRT1 obviously rescued MMP decrease caused by SIRT1 silencing (*p* < 0.01), verifying that HSYA could partially ameliorate si-SIRT1-mediated mitochondrial dysfunction.

### 3.9. HSYA Activates the SIRT1-FOXO3-BNIP3 Pathway to Promote Mitophagy in AC16 Cells

**Figure 9:**
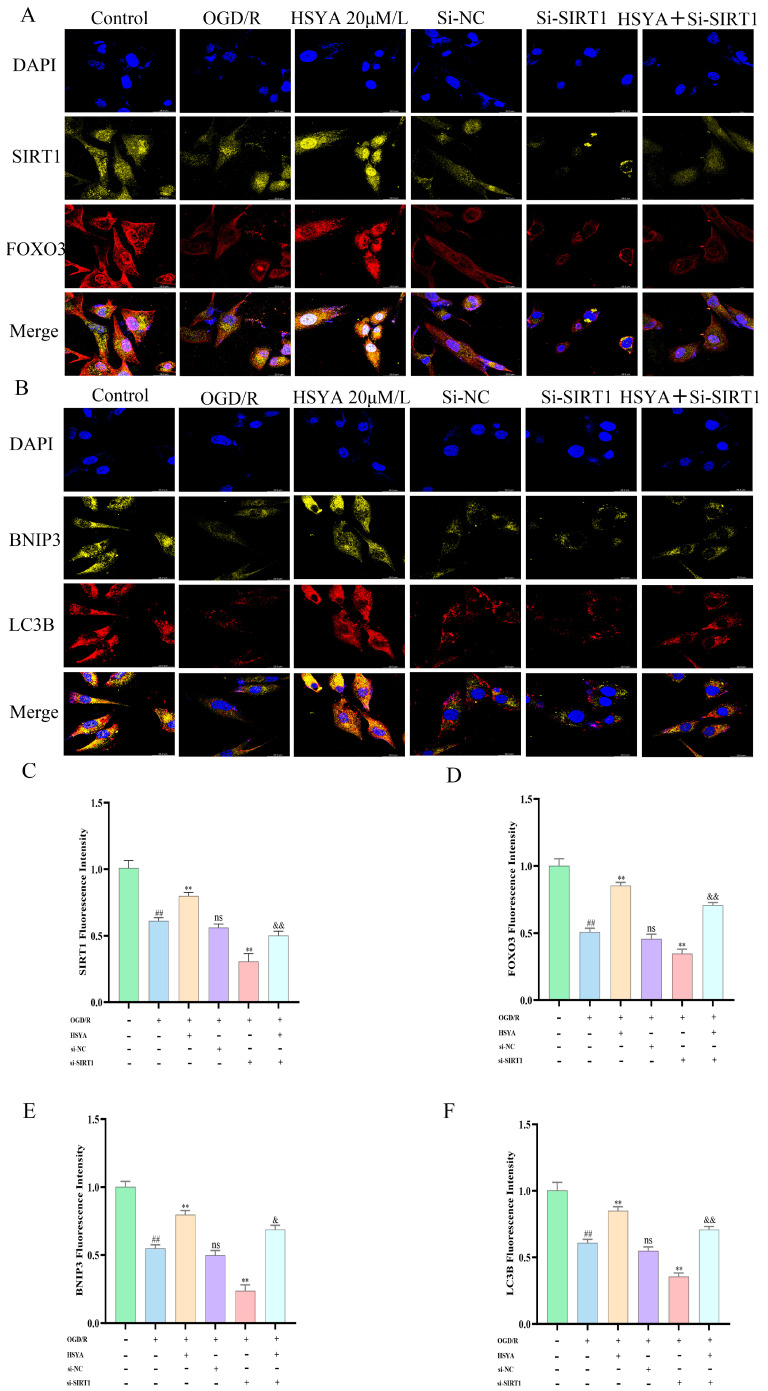
HSYA activates the SIRT1-FOXO3-BNIP3 signaling pathway to promote mitophagy. (**A**) Immunofluorescence staining showing the fluorescence intensity of SIRT1 and FOXO3 (*n* = 3). (**B**) Immunofluorescence staining showing the fluorescence intensity of BNIP3 and LC3B (*n* = 3). (**C**) Statistical analysis of SIRT1 fluorescence intensity. (**D**) Statistical analysis of FOXO3 fluorescence intensity. (**E**) Statistical analysis of BNIP3 fluorescence intensity. (**F**) Statistical analysis of LC3B fluorescence intensity. ^##^ *p* < 0.01 vs. control group; ** *p* < 0.01 vs. OGD/R group; ^&^ *p* < 0.05, ^&&^ *p* < 0.01 vs. HSYA 20 μmol/L group; ns = not significant.

Immunofluorescence double staining results showed that compared with the OGD/R group (model group), HSYA (20 μmol/L) treatment significantly increased the relative fluorescence intensities of SIRT1 and FOXO3 (both *p* < 0.01 vs. OGD/R group) (Figure 9A–C). Similarly, HSYA significantly enhanced the fluorescence intensities of BNIP3 and LC3B (both *p* < 0.01 vs. OGD/R group) (Figure 9D–F). In contrast, SIRT1 silencing (si-SIRT1 group) resulted in a marked decrease in the fluorescence intensities of SIRT1, FOXO3, BNIP3, and LC3B compared with the OGD/R group (all *p* < 0.01). Furthermore, HSYA administration on the basis of SIRT1 silencing significantly reversed the siRNA-induced downregulation of these target proteins (all *p* < 0.01 vs. si-SIRT1 group). These findings indicate that HSYA activates the SIRT1-FOXO3-BNIP3 signaling pathway and promotes mitophagy in AC16 cells, which is consistent with the immunofluorescence results from the in vivo experiments.

### 3.10. Effects of HSYA on Pathway Proteins and Autophagy-Related Proteins in AC16 Cells

**Figure 10:**
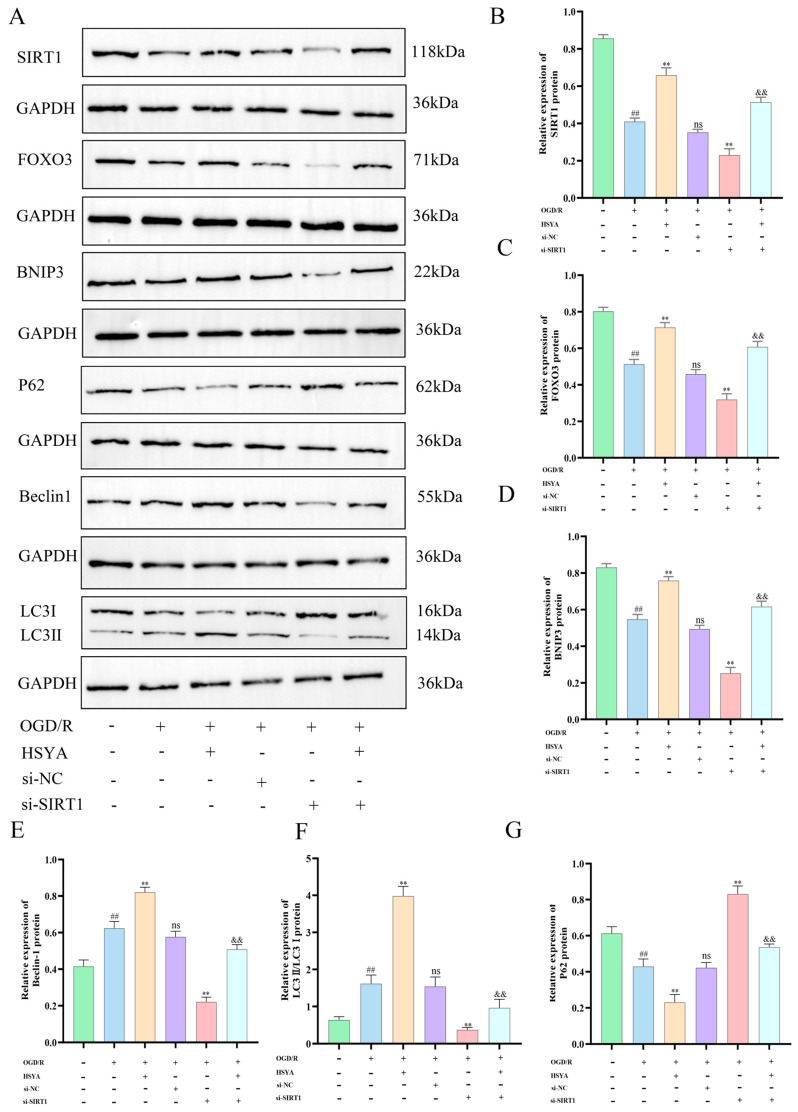
HSYA upregulates the expression of pathway-related proteins and autophagy-related proteins. (**A**) Western blot analysis of the protein expression of SIRT1, FOXO3, BNIP3, Beclin-1, P62, and LC3II/I (*n* = 3). (**B**) Densitometric analysis of SIRT1 protein bands. (**C**) Densitometric analysis of FOXO3 protein bands. (**D**) Densitometric analysis of BNIP3 protein bands. (**E**) Densitometric analysis of Beclin-1 protein bands. (**F**) Densitometric analysis of LC3II/I protein bands. (**G**) Densitometric analysis of P62 protein bands. ^##^ *p* < 0.01 vs. control group; ** *p* < 0.01 vs. OGD/R group; ^&&^ *p* < 0.01 vs. HSYA 20 μmol/L group.

Western blot (WB) results demonstrated that compared with the OGD/R group (Figure 10A), HSYA (20 μmol/L) treatment significantly upregulated the protein expression levels of SIRT1, FOXO3, and BNIP3 (all *p* < 0.01 vs. OGD/R group) (Figure 10B–D). In contrast, the si-SIRT1 group exhibited a marked downregulation in the expression of SIRT1, FOXO3, and BNIP3 (all *p* < 0.01 vs. OGD/R group). Notably, administration of HSYA to the si-SIRT1 group significantly reversed the siRNA-induced suppression of target protein expression (all *p* < 0.01 vs. si-SIRT1 group), suggesting that HSYA mediates mitophagy via the SIRT1-FOXO3-BNIP3 signaling pathway. Concurrently, HSYA treatment significantly increased the expression of the autophagy-related protein Beclin-1 and the LC3II/I ratio (both *p* < 0.01 vs. OGD/R group), while reducing P62 expression (*p* < 0.01 vs. OGD/R group)—indicating enhanced mitophagy activity. Compared with the OGD/R group, the si-SIRT1 group showed significantly decreased Beclin-1 expression and LC3II/I ratio (both *p* < 0.01) and elevated P62 levels (*p* < 0.01) (Figure 10E–G), reflecting attenuated autophagic activity. These in vitro findings are consistent with the results obtained from in vivo experiments.

### 3.11. Mechanism of HSYA in Regulating the SIRT1-FOXO3-BNIP3 Pathway in Ischemic Myocardial Tissue After MIRI and OGD/R-Induced AC16 Cells

**Figure 11:**
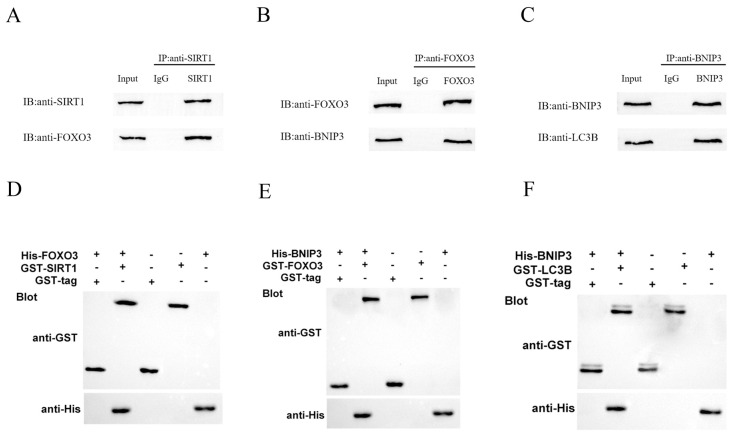
HSYA exerts its protective effects by promoting protein-protein interactions in the SIRT1-FOXO3-BNIP3 signaling pathway. (**A**–**C**) Co-immunoprecipitation (CO-IP) assays showing the interaction between SIRT1 and FOXO3, FOXO3 and BNIP3, and BNIP3 and LC3B, respectively. (**D**–**F**) Pull-down assays verifying the interaction between SIRT1 and FOXO3, FOXO3 and BNIP3, and BNIP3 and LC3B, respectively.

Co-immunoprecipitation (CO-IP) combined with Western blot (WB) analysis showed that FOXO3 protein was detected in the SIRT1 immunoprecipitates (Figure 11A), indicating a physical interaction between SIRT1 and FOXO3. Similarly, BNIP3 and LC3B proteins were identified in the immunoprecipitates using FOXO3 antibody-conjugated and BNIP3 antibody-conjugated Protein A/G magnetic beads, respectively (Figure 11B,C). These in vivo findings suggest that HSYA promotes mitophagy by facilitating the sequential interactions among SIRT1, FOXO3, and BNIP3, thereby attenuating myocardial ischemia-reperfusion injury in mice. In vitro experiments, pull-down assays were performed to validate these protein–protein interactions. Recombinant expression vectors GST-SIRT1 and His-FOXO3 were constructed, and proteins bound to GST-SIRT1-conjugated beads were detected using GST and His antibodies. Notably, FOXO3 was detected in the GST-SIRT1 bead precipitates (Figure 11D), confirming a direct physical interaction between SIRT1 and FOXO3. Similarly, recombinant vectors GST-FOXO3/His-BNIP3 and GST-BNIP3/His-LC3B were constructed (Figure 11E,F), and the pull-down results consistently verified the sequential interaction cascade (SIRT1-FOXO3-BNIP3-LC3B) observed in the in vivo CO-IP assay.

## 4. Discussion

MIRI is a pathological condition in which the degree of myocardial damage is further exacerbated following the relief of ischemic and hypoxic conditions and the restoration of blood perfusion [25,26], Early restoration of coronary blood flow is the primary strategy for clinical treatment of acute myocardial ischemia, mainly including percutaneous coronary intervention, thrombolysis, and coronary artery bypass grafting. However, blood flow reperfusion frequently triggers MIRI. Current conventional drugs merely improve coronary perfusion, stabilize atherosclerotic plaques, and regulate myocardial metabolism, while specific therapies targeting the pathogenic mechanisms of MIRI remain scarce. Accordingly, clinical drugs for targeted intervention against MIRI are still insufficient [27].

The occurrence of MIRI is often accompanied by mitochondrial damage. Mitochondrial quality control is crucial in this process. Mitochondrial biogenesis and mitophagy are key processes for maintaining mitochondrial quality. Studies have indicated that HSYA regulates SIRT1 to activate the PGC-1α pathway and further promote mitochondrial biogenesis [28]. Mitochondrial autophagy, in contrast, is a process that alleviates mitochondrial dysfunction, improves mitochondrial quality, and maintains cardiac homeostasis by degrading abnormal or damaged mitochondria [29]. Studies have demonstrated that mitochondrial autophagy can regulate cardiovascular activities through diverse pathways and thereby participate in the pathophysiological processes of cardiovascular diseases such as heart failure [30,31]. Therefore, timely correction of mitochondrial function and enhancement of myocardial mitochondrial autophagy after the onset of MIRI are of crucial importance for alleviating myocardial injury and restoring cardiac function.

In the untargeted metabolomics analysis of samples from the sham operation group and MIRI group, our study revealed that mitochondrial autophagy was highly correlated with the FOXO3 pathway [16]. FOXO3 (Forkhead box O3) is a core member of the Forkhead box O (FoxO) family and also a key node molecule in the cellular autophagy regulatory network. Its function in autophagy is mainly manifested in the transcriptional regulation of autophagy-related gene expression, thereby participating in the selective autophagy process, and its functional activity is precisely regulated by SIRT1, an upstream signaling pathway [32]. SIRT1 is a member of the sirtuin (SIRT) family, which is widely expressed in various somatic and germ cells and involved in multiple physiological processes including cell proliferation, differentiation, metabolism, and apoptosis [33]. By directly binding to its downstream target FOXO3, SIRT1 activates the SIRT1-FOXO3 axis, resulting in an increased protein expression level of FOXO3 [34,35]. Meanwhile, as a key effector molecule of autophagy, FOXO3 can bind to the BNIP3—a receptor closely associated with mitochondrial autophagy. Under ischemic and hypoxic conditions, BNIP3 is transcriptionally regulated by upstream FOXO3, with its expression level upregulated to mediate mitochondrial autophagy, thus facilitating the occurrence of mitochondrial autophagy and cell apoptosis. In response to cellular hypoxia or pathological damage, SIRT1 activates FOXO3, which in turn mediates the upregulation of BNIP3 protein expression, thereby promoting the initiation of mitochondrial autophagy in cardiomyocytes [36]. In addition, BNIP3 acts as an inducer receptor that directly interacts with microtubule-associated protein 1 light chain 3 (LC3) via its conserved LC3-interacting region (LIR) motif, thereby selectively targeting mitochondria for autophagosomal degradation. Damaged mitochondria bind to autophagic vesicles, are engulfed into the vesicles, and then transported out of the cells [37]. In addition, previous studies on the SIRT1-FOXO3-BNIP3 axis have mainly focused on tumors, nerve injury, liver injury, and other stress models. Its regulatory role in mitophagy has been partially reported, whereas whether this pathway is involved in HSYA-mediated intervention against MIRI remains poorly elucidated. Based on previous metabolomics studies conducted by our research group and combined in vivo MIRI mouse models and in vitro OGD/R-stimulated AC16 cardiomyocyte models, the present study confirmed that HSYA could upregulate the expression levels of SIRT1, FOXO3, BNIP3, and LC3B and strengthen the protein interactions between SIRT1-FOXO3, FOXO3-BNIP3, and BNIP3-LC3B. Further experiments using the SIRT1 inhibitor EX527 and si-SIRT1 revealed that pharmacological inhibition or genetic knockdown of SIRT1 markedly abrogated the protective effects of HSYA on mitochondrial function, the expression of autophagy-related proteins, as well as myocardial and cellular injury. Collectively, these findings indicate that the SIRT1-FOXO3-BNIP3 signaling pathway may serve as a core mechanism by which HSYA modulates mitophagy during MIRI progression.

HSYA is the core bioactive component of *Carthamus tinctorius* L. (safflower). Its efficacy in promoting blood circulation to remove blood stasis is not only a classic characterization of safflower’s medicinal value in traditional Chinese medicine (TCM) theory but also fully validated by modern pharmacology [38]. According to TCM theory, safflower is pungent in flavor and warm in nature, with meridian tropism in the heart and liver meridians. The pungent property facilitates stasis dispersion, while the warm property promotes meridian dredging, thus rendering safflower highly effective in treating blood stasis syndrome; HSYA serves as the core material basis for this therapeutic efficacy [39]. Compared with conventional clinical therapies, it can block injuries originating from mitochondrial metabolism and simultaneously exert antioxidant, anti-inflammatory, anti-apoptotic, and vascular-protective effects. It avoids combined medication, with high safety profiles. Additionally, it ameliorates myocardial remodeling and reduces disease recurrence risk, making it a superior candidate for the prevention and treatment of MIRI [40]. Our previous findings have demonstrated that HSYA exerts a prominent protective effect against ischemic myocardial injury, such as inhibiting ferroptosis and promoting myocardial angiogenesis [15,16]. In terms of concomitant cardiac-cerebral treatment, HSYA exerts a therapeutic effect on stroke-heart syndrome (SHS) by activating the ZRP1-NLRP3 signaling pathway [41]. These findings are consistent with the present study, confirming that HSYA exerts definite tissue-protective effects against ischemia/reperfusion injury. Unlike previous studies that merely focused on the ameliorative effects of HSYA on myocardial injury phenotypes, the current study further explored its underlying mechanism from the perspectives of mitochondrial quality control and mitophagy. The present results demonstrated that HSYA not only reduced myocardial infarct size, improved cardiac function, and decreased the levels of myocardial injury markers including LDH, CK-MB, and cTnI in MIRI mice, but it also ameliorated aberrant mitochondrial ultrastructure and mitochondrial membrane potential as well as modulated the expression of autophagy-related proteins such as LC3II/I, Beclin-1, and P62.

The present study did not merely focus on the improvement of myocardial injury phenotypes induced by HSYA but further elucidated its underlying mechanism from the perspectives of mitochondrial quality control and mitophagy. Collectively, these findings suggest that the cardioprotective effects of HSYA against MIRI are closely associated with the elimination of damaged mitochondria and the maintenance of mitochondrial homeostasis and reveals that HSYA may alleviates myocardial ischemia-reperfusion injury by activating the SIRT1-FOXO3-BNIP3 signaling pathway to promote mitophagy, thereby identifying a novel therapeutic target for resisting MIRI.

## 5. Conclusions

In this study, we comprehensively employed untargeted metabolomics analysis, small interfering RNA (siRNA)-mediated specific gene silencing technology, and combined them with imaging and morphological detection assays as well as techniques including co-immunoprecipitation (CO-IP) and pull-down assay. On this basis, we systematically elucidated the underlying mechanism of HSYA against MIRI in a multi-level manner, spanning from the molecular and cellular levels to the whole-animal level. this study demonstrates that HSYA effectively promotes mitophagy in mice and ameliorates MIRI by activating the SIRT1-FOXO3-BNIP3 signaling pathway. These findings provide novel insights into the molecular mechanism underlying MIRI treatment, uncover the clinical potential of HSYA for MIRI management, and offer new therapeutic strategies and approaches for the treatment of MIRI. However, the results of the present study are mainly based on the mouse MIRI model and the OGD/R-injured AC16 cell model, which remain at the experimental model stage and cannot fully reflect the complex pathological conditions of clinical patients with MIRI. As the detection of mitochondrial flux is lacking, future studies using specific mitophagy reporters and additional models will be necessary to fully confirm these mechanisms, which also clarifies the future research direction of this experiment. The clinical efficacy, safety, optimal dosage, and administration timing of HSYA still need to be further verified by pharmacokinetic studies, toxicological tests, large animal experiments, and clinical trials.

## Data Availability

The original contributions presented in this study are included in the article/Supplementary Material. Further inquiries can be directed to the corresponding authors.

## Supplementary Materials

The following supporting information can be downloaded at https://www.mdpi.com/article/10.3390/nu18111780/s1, Figure S1. Four-color immunofluorescence staining under HPF.
