# Supplementary figures and images for: Hydroxysafflor Yellow A Regulates SIRT1-FOXO3-BNIP3 Signaling Pathway to Promote Mitophagy: A Novel Therapeutic Strategy for Myocardial Ischemia-Reperfusion Injury
Source: Nutrients. 2026 May 31;18(11):1780. doi: 10.3390/nu18111780 (PMC13259012; doi:10.3390/nu18111780)

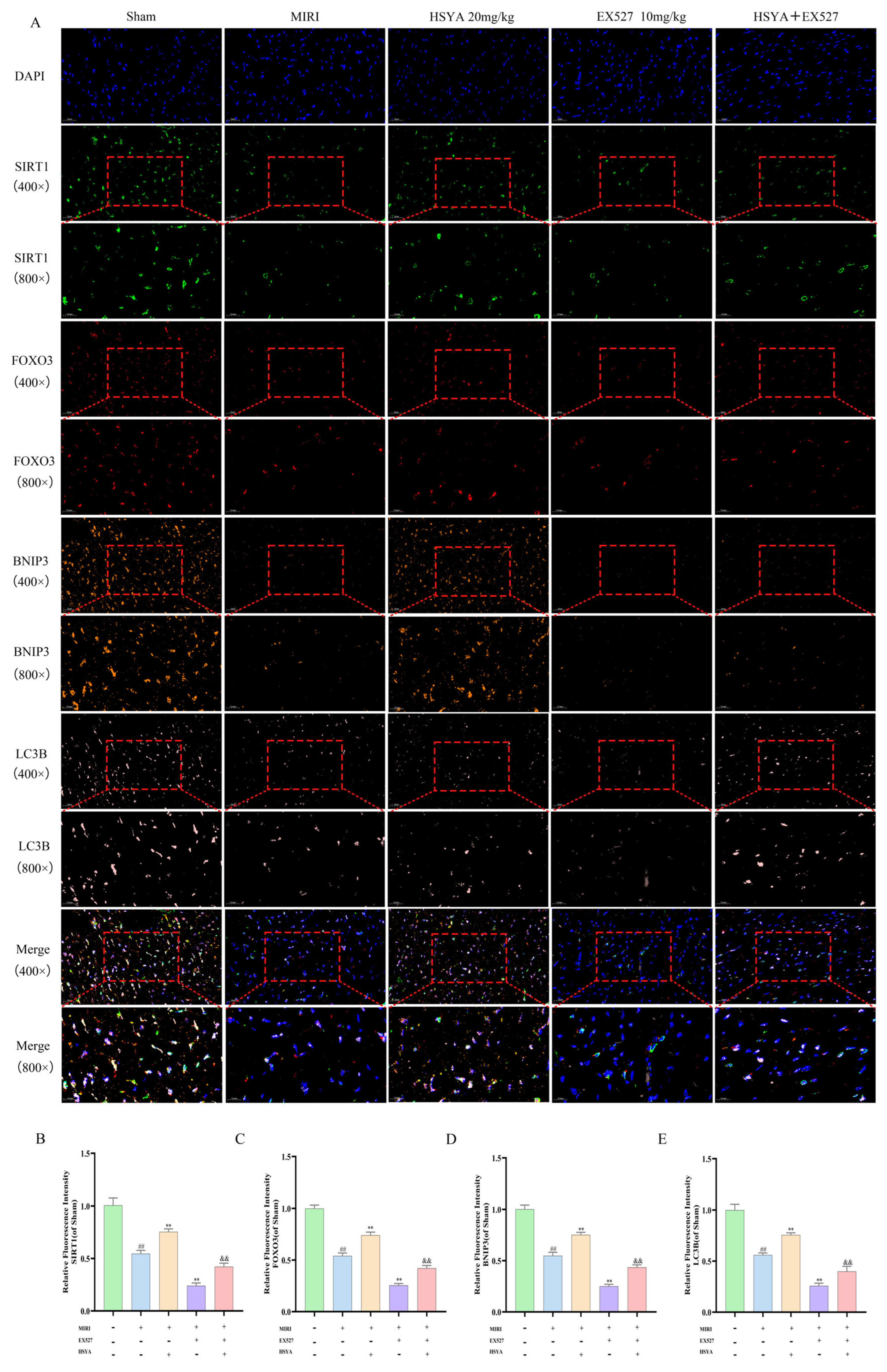

Supplement: Supplementary file 1 [file nutrients-18-01780-s001.zip › nutrients-4303402-supplementary.tif]
